# Novel Approach for the Surgical Management of Nonunion After Minimally Invasive Bunionectomy Using Bone Harvesting and Bioabsorbable Screw Fixation: A Case Report

**DOI:** 10.7759/cureus.97059

**Published:** 2025-11-17

**Authors:** Samantha Reed, Joseph Torkieh, Vishwa Thakor, Jay Bhuta, Rahul Mittal

**Affiliations:** 1 Podiatry, Cooperman Barnabas Medical Center, Livingston, USA; 2 Medicine, Rutgers Robert Wood Johnson Medical School, New Brunswick, USA; 3 Health Informatics, Rutgers University, New Brunswick, USA

**Keywords:** autologous bone grafting, bioabsorbable screw fixation, bone augmentation, bony callus formation, bunion correction, hallux valgus surgery, minimally invasive bunionectomy, nonunion, surgical fixation, surgical revision

## Abstract

Hallux valgus is a common forefoot deformity that can be surgically corrected with minimally invasive bunionectomy, which offers advantages such as smaller incisions, reduced soft tissue trauma, and shorter recovery. Despite these benefits, nonunion remains a challenging complication with limited consensus on optimal management. We report the case of a 42-year-old woman with situs inversus totalis who presented with painful left hallux abducto valgus unresponsive to conservative therapy. She underwent a minimally invasive bunionectomy, but at seven months, she continued to have persistent pain, and radiographic findings confirmed nonunion. Revision surgery was performed using autologous tibial bone grafting, removal of cannulated screws, and fixation with two bioabsorbable screws. Radiographs obtained one month postoperatively demonstrated early bony callus formation, and at three months, robust callus with reduced radiolucency indicating osseous union. Clinically, the patient experienced improved pain, functional recovery, and no hardware-related irritation. This case highlights the potential role of bioabsorbable screw fixation combined with autologous bone grafting as an effective strategy for treating nonunion following minimally invasive bunionectomy, offering the dual benefits of enhanced healing and avoidance of long-term hardware complications.

## Introduction

Hallux valgus, commonly referred to as a bunion, is a progressive forefoot deformity characterized by lateral deviation of the hallux and medial deviation of the first metatarsal. This misalignment often results in pain, structural instability, and difficulty with footwear use [1,2]. In symptomatic cases, surgical correction via bunionectomy, often a first distal metatarsal osteotomy, remains the standard of care. Both open and minimally invasive surgery (MIS) techniques are employed based on patient- and surgeon-specific factors [3,4].

MIS bunionectomy offers potential advantages over open techniques, including reduced soft tissue disruption, smaller incisions, and shorter recovery times. However, complications such as recurrence, stiffness, hardware irritation, and notably, nonunion at the osteotomy site can impair functional outcomes and patient satisfaction [3-5].

Bone nonunion is defined as the failure of osseous healing within an expected time frame, typically between six to eight months, accompanied by persistent pain, instability, or functional limitation [1]. While relatively uncommon after bunion surgery, nonunion poses a significant clinical challenge due to the biomechanical demands of the forefoot, limited vascularity, and the technical difficulty of achieving stable fixation in small bone segments [6]. Nonunions are generally classified into hypertrophic (biologically active blood supply but mechanically unstable), atrophic (poor blood supply and unstable), and oligotrophic (combination of both). This classification guides treatment approaches aimed at correcting mechanical instability, enhancing biological healing, or both [6].

Numerous systemic and local risk factors contribute to nonunion. Systemic factors include advanced age, smoking, diabetes mellitus, osteoporosis, malnutrition, and peripheral vascular disease [7]. Local contributors such as infection, inadequate stabilization, compromised perfusion, and soft tissue injury are particularly impactful in foot surgery, where the interplay between mechanical stress and limited bone size requires precise technique [8]. While MIS reduces soft tissue trauma, it may also increase the technical complexity of osteotomy alignment and fixation [3,9].

In cases of confirmed nonunion, revision surgery is often indicated. Conventional fixation methods use non-absorbable implants such as titanium or stainless steel, which are effective but may cause long-term irritation or imaging artifacts or require removal. Consequently, bioabsorbable fixation devices have gained popularity [10]. Composed of materials such as polyglycolide or polylactide, these absorbable implants provide temporary mechanical support and gradually degrade over time, minimizing long-term complications. Studies on absorbable implants in ankle fracture fixation report outcomes comparable to metal hardware in terms of radiographic healing and functional recovery, while offering advantages such as reduced implant palpability and lower reoperation rates. Importantly, no significant differences have been noted in rates of infection, pain, osteoarthritis, or foreign body reactions [11,12].

This case report describes a novel approach to managing nonunion following a bunionectomy, utilizing a combination of bone augmentation and bioabsorbable screw fixation. It highlights intraoperative decision-making, surgical technique, and postoperative outcomes, contributing to the growing body of evidence supporting bioabsorbable implants in forefoot surgery.

## Case presentation

A 42-year-old woman with situs inversus totalis presented with painful left hallux abducto valgus. She had a remote history of right hallux fracture in adolescence involving the growth plate, followed by a right bunionectomy in 2012. The postoperative course of the right foot was prolonged, complicated by persistent pain, stiffness, and hardware removal. Importantly, she developed avascular necrosis of the first metatarsal head after an open distal metatarsal osteotomy, which prompted her preference for an MIS approach on the contralateral side.

At presentation, she described worsening pain over the medial aspect of the left first metatarsophalangeal joint and noted a recurrent cyst in the same region. The cyst had previously been aspirated, with residual bruising and discomfort.

Imaging and diagnostic workup

**Figure 1:**
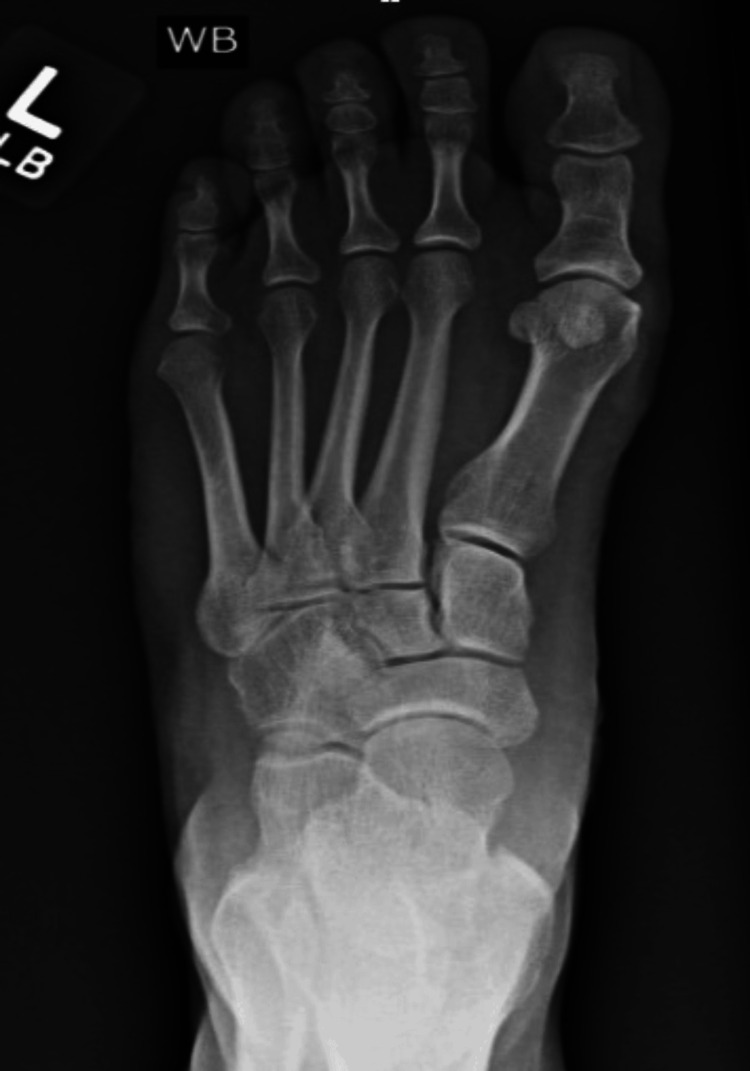
Preoperative weight-bearing radiograph demonstrating left hallux valgus deformity

Weight-bearing radiographs of the left foot demonstrated a hallux valgus deformity (Figure 1). A small ganglion was noted adjacent to the medial aspect of the distal first metatarsal, near the anticipated osteotomy site. After failing conservative management, including footwear modification and cyst aspiration, the patient elected to proceed with surgical intervention after being counseled on the risks, alternatives, and potential complications.

Management and surgical intervention

The patient underwent a minimally invasive left bunionectomy. A preoperative nerve block was administered to the left lower extremity, and the patient was placed under general anesthesia in the supine position. A pneumatic thigh tourniquet was applied but remained deflated throughout the procedure. Following sterile preparation and draping, a 1 cm stab incision was made over the medial first metatarsal neck. A transverse osteotomy was performed using a side-cutting burr, and the metatarsal head was translated laterally into corrected alignment. Temporary fixation was achieved with K-wires, after which two headless cannulated screws were inserted under fluoroscopic guidance. Intraoperative imaging confirmed appropriate screw positioning and deformity correction. The medial bone shelf was removed with the burr, the site irrigated with sterile saline, and the wound closed in layers using 5-0 Monocryl. The incision was dressed with betadine-soaked aseptic gauze, Kerlix, and an ACE wrap. No intraoperative complications occurred.

Postoperative course

The patient was instructed to remain non-weight-bearing for two weeks, then transition to progressive weight-bearing in a controlled ankle motion (CAM) boot with physical therapy.

**Figure 2:**
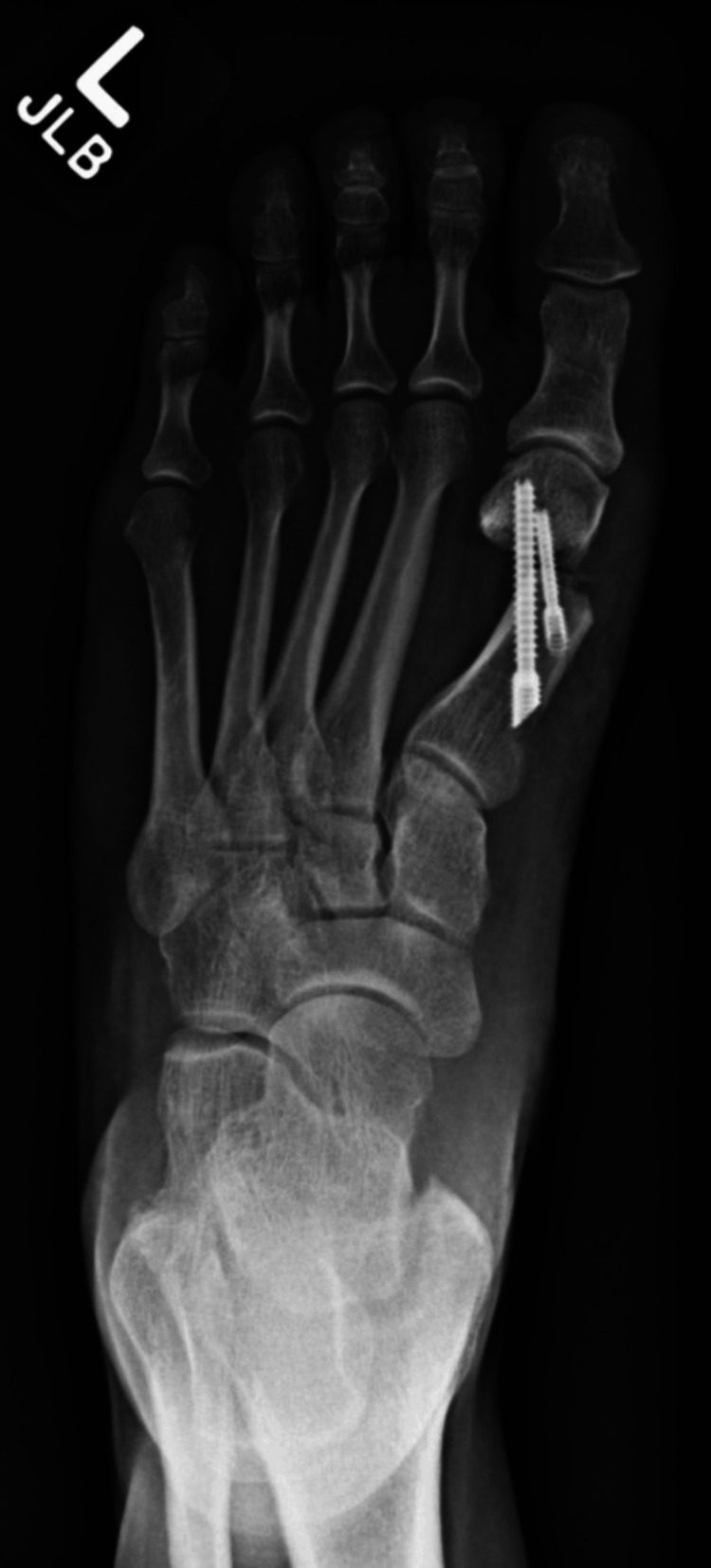
Radiograph two weeks after minimally invasive bunionectomy showing satisfactory alignment and hardware placement

At two weeks, radiographs demonstrated satisfactory alignment and hardware position (Figure 2). However, during this period, she reported performing calf raises against medical advice, which likely caused cracking of the dorsal cortex of the first metatarsal and subsequent hardware stress.

**Figure 3:**
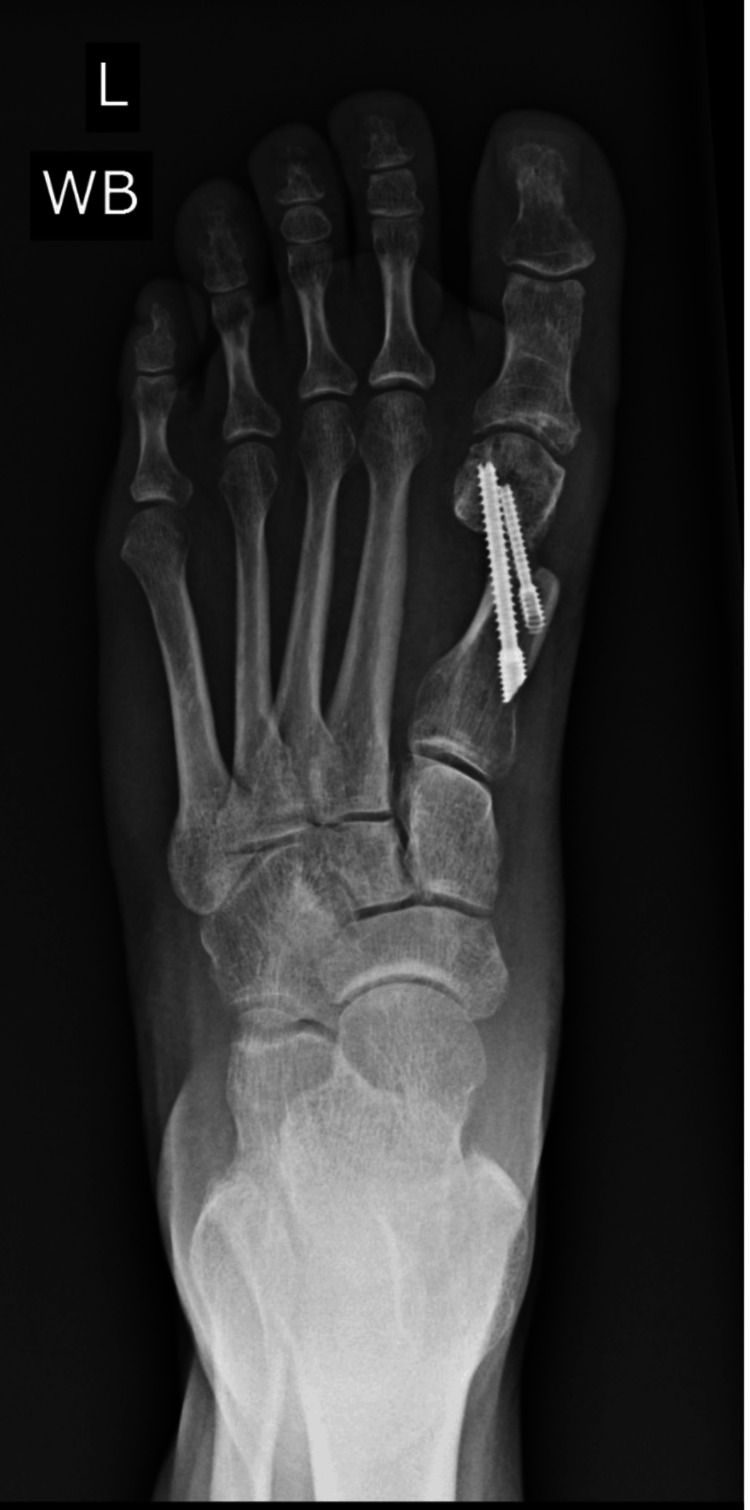
Radiograph five months postoperatively demonstrating persistent radiolucency at the osteotomy site, consistent with nonunion

**Figure 4:**
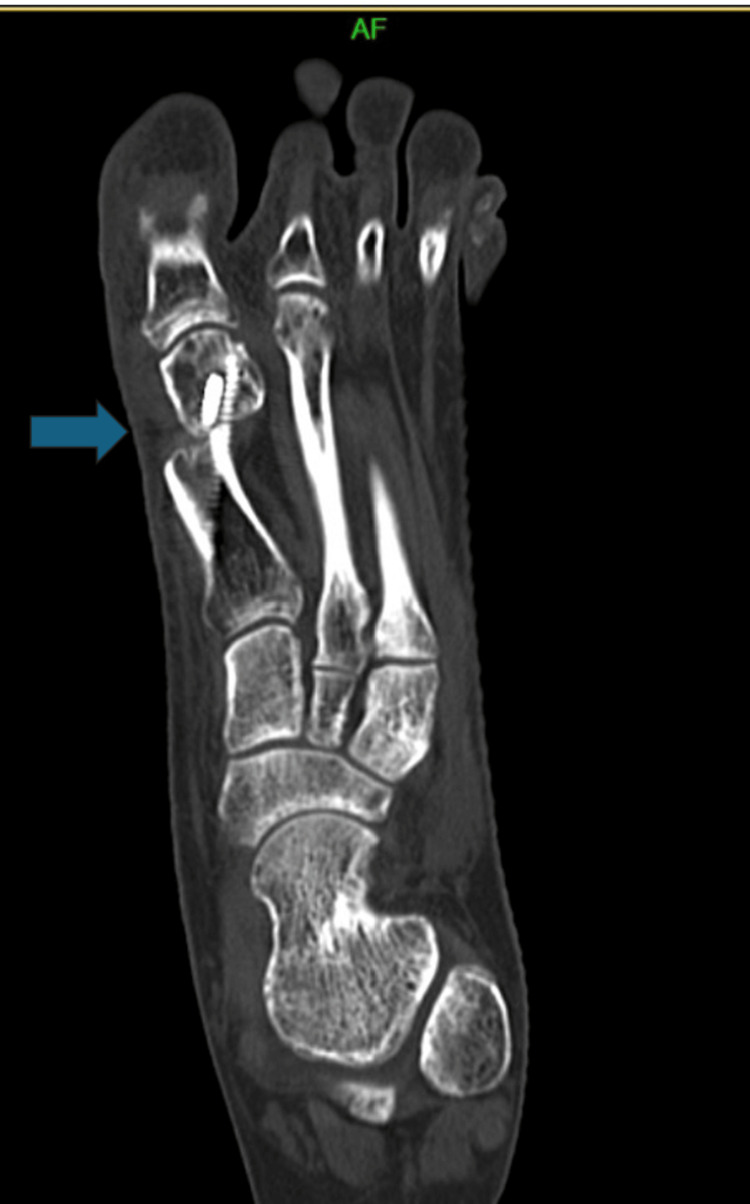
Coronal CT image confirming absence of osseous union across the osteotomy site (blue arrow), indicating the persistent gap at the osteotomy margins

However, serial imaging up to six months revealed persistent radiolucency at the osteotomy site without callus formation (Figure 3). CT imaging confirmed nonunion (Figure 4). Clinically, she reported ongoing pain at the incision site and required continued CAM boot use even at 19 weeks.

**Figure 5:**
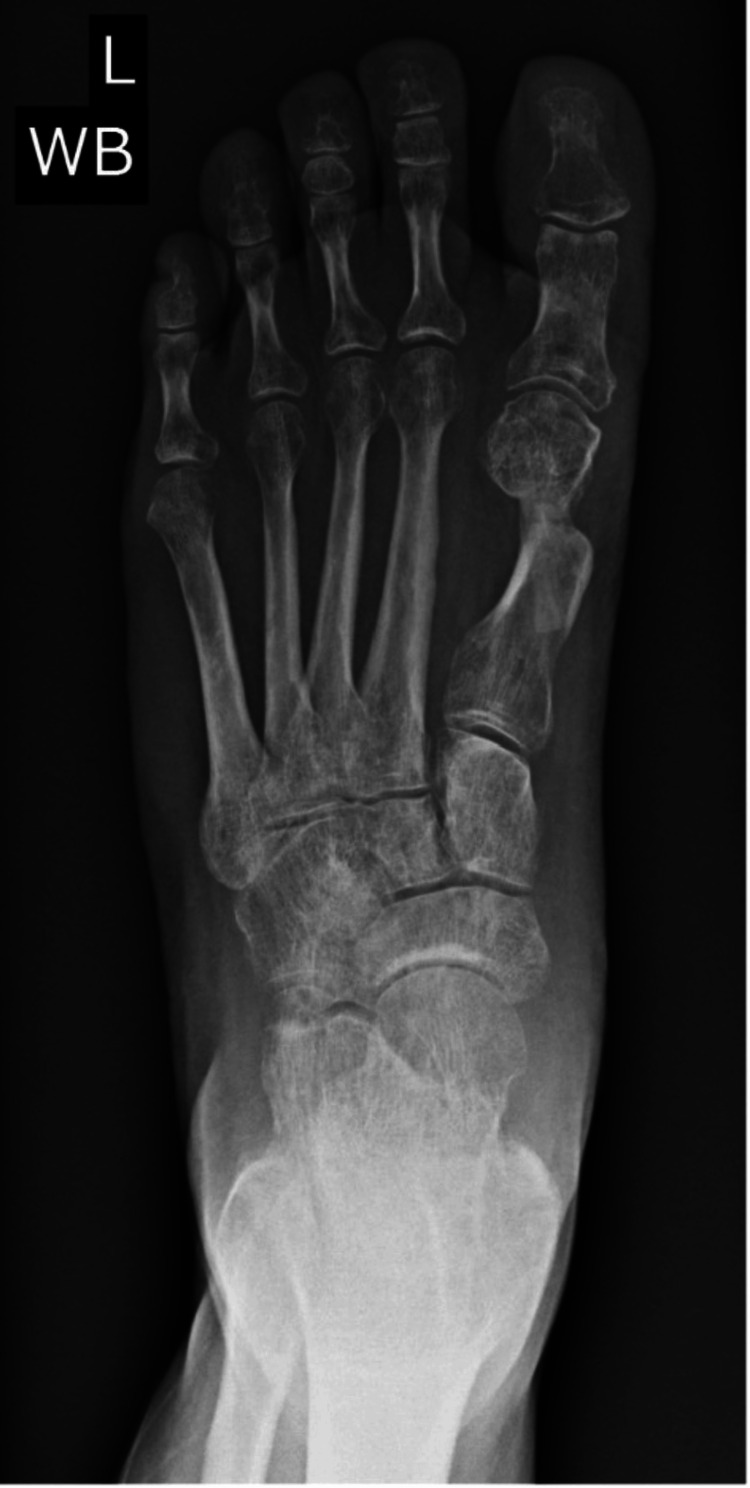
Radiograph three months after revision surgery showing robust callus formation, decreased radiolucency, and progressive osseous union

**Figure 6:**
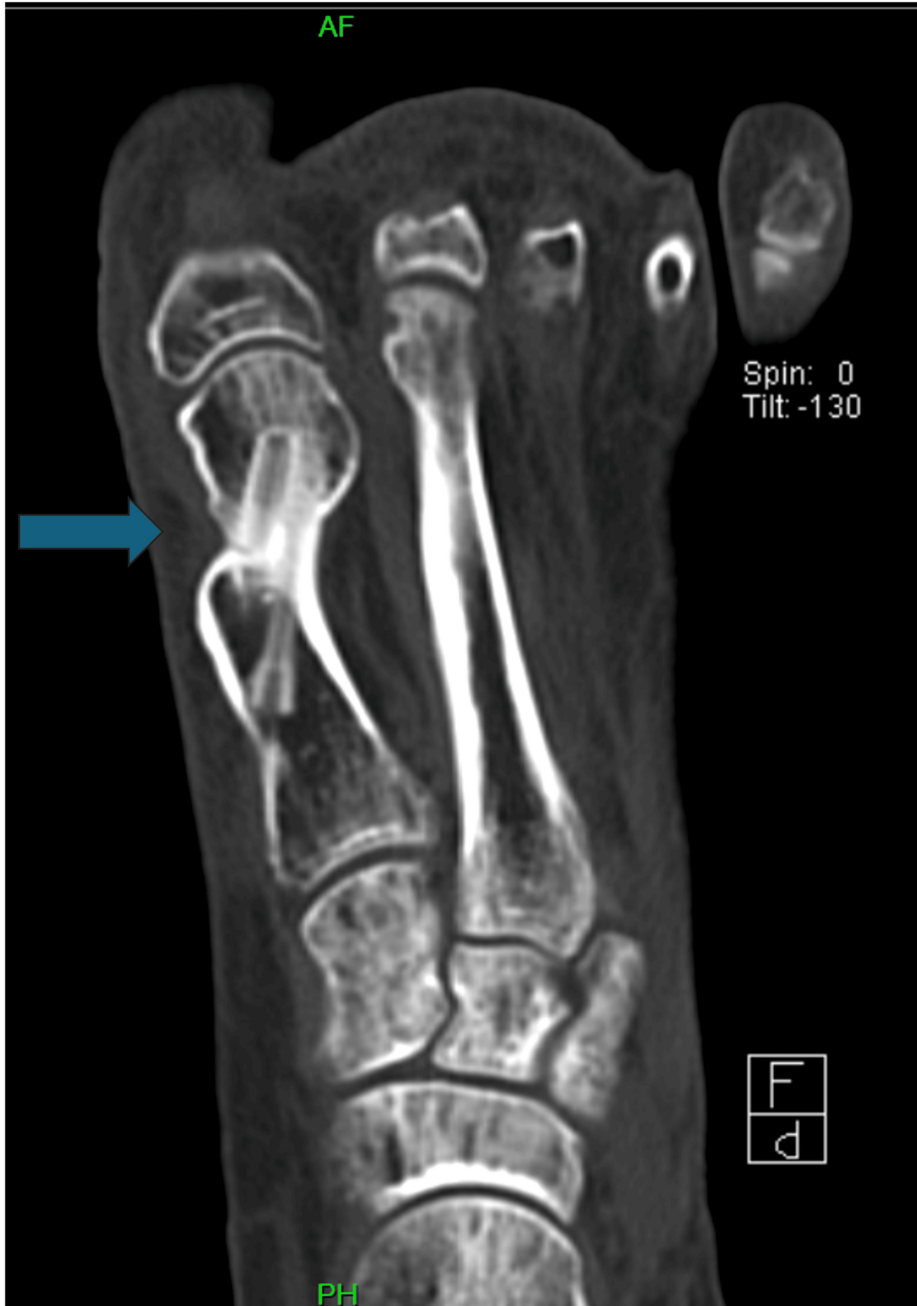
Coronal CT image confirming osseous union at three months post-revision (blue arrow), indicating the site of bone healing

Given radiographic and clinical findings consistent with nonunion, revision surgery was performed. The procedure involved harvesting an autologous tibial bone graft, removing the existing cannulated screws, and securing the osteotomy with two bioabsorbable magnesium screws. At three months post-revision, follow-up radiographs and CT imaging demonstrated robust callus formation, reduced radiolucency, and progressive union at the osteotomy site, consistent with successful osseous healing (Figure 5, Figure 6).

## Discussion

Successful management of nonunions requires a comprehensive understanding of both the biological foundations of fracture repair and the emerging field of osseoimmunology. The interaction between bone cells and the immune system plays a pivotal role in the fracture healing cascade. The Diamond Concept of fracture healing underscores the need to balance biological and mechanical factors for optimal outcomes. This framework emphasizes the availability of osteogenic cells, osteoconductive scaffolds, osteoinductive factors, and a favorable mechanical environment, all of which can be targeted to enhance bone regeneration, particularly in the setting of nonunion. The integration of biological therapies with stable fixation represents a promising strategy to achieve predictable union.

Recent advances in bioabsorbable fixation devices provide additional options for optimizing healing. Biodegradable magnesium screws (Magnezix) have demonstrated postoperative outcomes equivalent to titanium screws in hallux valgus surgery, with significant improvements in pain, function, and overall quality of life [13]. The unique advantage of magnesium-based implants lies in their gradual biodegradation, eliminating the need for secondary hardware removal and thus reducing the risks of additional surgery, infection, and scarring, while lowering healthcare costs. Unlike polymer-based screws, magnesium undergoes corrosion rather than hydrolytic degradation, avoiding the inflammatory responses sometimes observed with polymeric implants. Radiographic follow-up has demonstrated complete resorption of these screws within two to three years, with replacement by native bone and no adverse sequelae [13]. Furthermore, the biomechanical properties of magnesium, with a modulus of elasticity closer to bone, minimize stress shielding and promote physiologic load transfer, thereby enhancing osteoconductive healing.

In cases with critical bone loss, biological augmentation is often necessary. Autologous bone graft remains the gold standard, but allograft options, particularly demineralized bone matrix (DBM), have gained traction as adjuncts to expedite healing. DBM contains bioactive proteins that stimulate osteogenesis and may shorten the time to union, particularly in patients with risk factors for delayed healing [14]. A retrospective cohort analysis of 72 patients (77 feet) undergoing third-generation minimally invasive hallux valgus correction compared outcomes between patients receiving DBM gel (n = 25) and controls (n = 52) [14]. While both groups achieved 100% union with no malunions or nonunions, the DBM group demonstrated a shorter mean time to union (3.52 ± 1.58 months vs. 4.36 ± 2.20 months). Although the difference did not reach statistical significance (p = 0.09), the trend toward faster union suggests clinically meaningful benefits, particularly for smokers, diabetics, and osteoporotic patients. Importantly, augmentation with DBM was not associated with increased complications such as infection, immune reactions, or recurrence [14].

Taken together, the integration of bioabsorbable magnesium screws and biological augmentation represents a compelling alternative to conventional approaches in minimally invasive foot surgery. By combining mechanically favorable, osteoconductive implants with biologically active graft materials, surgeons can potentially reduce healing time, avoid secondary procedures, and promote more physiologic bone regeneration. This case highlights the importance of individualized strategies in addressing complications such as nonunion, reinforcing the need for a biologically informed, mechanically sound, and patient-centered approach in modern foot and ankle surgery.

While this case demonstrates successful early consolidation following revision surgery, the follow-up period is limited to three months after the use of bioabsorbable magnesium screws and autologous bone grafting. Longer-term follow-up will be important to evaluate the durability of the correction and to confirm the expected two- to three-year resorption profile of magnesium-based implants [13]. The patient will continue to be monitored longitudinally, and future imaging is anticipated to demonstrate complete implant resorption and maintenance of osseous stability.

## Conclusions

This case demonstrates that the combined use of bioabsorbable screw fixation and autologous bone grafting can be an effective strategy for managing nonunions in minimally invasive foot surgery. Radiographic follow-up confirmed progressive consolidation, highlighting the importance of regular imaging for assessing healing and guiding management. By providing stable fixation while gradually resorbing, bioabsorbable implants eliminate the need for secondary hardware removal and may support biologically integrated repair, thereby improving patient outcomes and reducing overall treatment burden. Future prospective and comparative studies are necessary to establish the relative advantages of bioabsorbable fixation over traditional methods and to develop evidence-based protocols for biological augmentation in high-risk patients.
